# Leveraging the Knowledge of Our Peers: Online Communities Hold the Promise to Enhance Scientific Research

**DOI:** 10.1371/journal.pbio.0040199

**Published:** 2006-06-13

**Authors:** Thomas J Sharpton, Arpan A Jhaveri

## Abstract

SIPHS is a tool that leverages scientific resources online in a different fashion: rather than searching for online documents, users search for community members with a particular knowledge set.

Without knowledge to contextualize an observation, scientific discovery becomes guesswork. Simple facts may make or break a theory, but without these vital bits of information, theory remains theory. Because access to information is crucial to the formation of reasonable hypotheses and the proper interpretation of data, tools that expedite information retrieval and simplify information exchange are of great importance to scientists.

Indeed, many researchers are well aware of the Internet's influence on our ability to locate and access specific knowledge: online journal publications and searchable databases such as the NCBI's PubMed have significantly expedited information acquisition. Rather than combing through library stacks for hours on end, a few keystrokes and mouse-clicks are all it takes to identify a wealth of knowledge on a particular subject. But all tools have their limitations. Conventional search engines such as PubMed provide access to information but prohibit users from interacting with the source of knowledge and clarifying the underlying context. Frequently, papers will contain information of interest but require substantial background reading (and hence additional searching) to properly contextualize the information for critical analysis. What if, rather than receiving information from a static entity in response to a limited query, we could dynamically interact with the source to mold the response to cater to our specific research needs?

An online revolution is changing the way we think about obtaining information. By facilitating interaction between users in an online community, new tools harness the collective wisdom of their participants to identify and critically review information. Rather than simply reading published encyclopedia material, Wikipedia ( http://www.wikipedia.org), for example, allows users to add and amend information in its encyclopedia, creating a dynamic ever-changing document subject to the review of the community. An entry by one user may subsequently spawn an additional entry by another user on the same subject. These community-based tools, which have existed outside the scientific domain for some time now, have great potential to enhance research by improving the ability to share scientific information online.

For example, Connotea ( http://www.connotea.org), developed by the Nature Publishing Group, is a reference management and social bookmarking tool. When researchers come across articles of interest, they can bookmark the article on their Connotea account and apply descriptive identifiers, known as tags, to the article for organizational purposes. Other users can then search for tags (such as “avian flu” or “SH2 domain”) and see what their peers have bookmarked as being relevant online information for the subject at hand. Because other members have already determined which online sources of information are important for a given subject (by taking the time to bookmark the item), users new to the subject need to spend less time searching for valuable information. When new information is added to the community database, all members subsequently benefit.

We have developed SIPHS ( http://www.siphs.com), a tool that leverages an online community in a different fashion: rather than searching for online documents, users search for community members with a particular knowledge set. We established SIPHS in response to a shared frustration. The Internet was designed to put people in touch, but it is quite difficult to identify individuals that possess very specific, often highly technical knowledge. [Fig pbio-0040199-g001]

**Figure pbio-0040199-g001:**
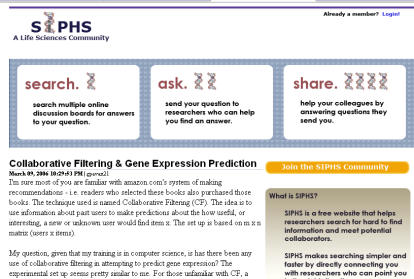
Members of SIPHS ( http://www.siphs.com) can search for peer-generated information, ask questions of other members, and provide peer support

The SIPHS community is currently comprised of more than 200 biology and biomedical researchers spread across 30 countries. Members of the community are tagged with their respective areas of expertise, and queries for information are submitted via an electronic message to experts in relevant fields. By enabling direct communication with knowledgeable and experienced individuals, refining searches becomes easier (as searching is no longer keyword dependent), background information is more quickly clarified, and new ideas are more rapidly spawned. In essence, this mimics the offline world in that the best source of information is often a colleague who has experience with the problem at hand. SIPHS is self-funded and, like all the tools mentioned in this discussion, free to use.

Other tools allow users to identify what other researchers are saying about particular pieces of information. Postgenomic ( http://www.postgenomic.com), for example, identifies comments made on life science blogs that are pertinent to particular journal papers. Users search for a paper of interest and are returned a collection of reviews on the particular paper made by the life science blogging community. Authors can view what others are saying about their publications, and researchers can get an additional side of a paper's story. Often, hearing what a peer has to say on a particular subject can help piece together a puzzle or form a critical opinion.

Finally, other tools utilize a community of experts to curate database information. As an example, consider the *Neurospora crassa* Community Annotation Project (CAP) ( http://www.broad.mit.edu/annotation/genome/neurospora/CAHome.html), designed by the Broad Institute. Gene annotation is a critical step in transforming a genome sequence into a useful biological research tool, but annotation is difficult and time consuming. By allowing experts in the *Neurospora* community to evaluate and amend the annotation data for the organism, the CAP adds an extra layer of evaluation to the database and accelerates the annotation process. It may very well be that this type of genome annotation method becomes a standard among sequencing centers in the near future.

Community-based tools have the potential to revolutionize access to scientific information but, like all tools, are not without their limitations. First, the information obtained by community tools is subject to the biases of the users generating the information. As a result, we must continue to critically evaluate the information received from these tools. Fortunately, community members often, through civil discourse or amendments, provide checks on bad or incorrect information coming from other members, minimizing these types of problems. Second, these tools are not designed to replace conventional search engines. We should be clear that search engines provide a great utility to the scientific community and certainly have their place in research, especially when probing a new subject for ideas.

The most fundamental limitation is that the utility of these sites is a direct function of the level of community participation. This is, perhaps, the greatest rate-limiting step: for some reason or another, scientists have been reluctant to join and participate in virtual communities. We believe this problem is a result of the unfamiliarity and relative novelty of community-based tools in the scientific domain, and are hopeful that by exposing information regarding their existence, researchers will be more willing to adopt them into their research repertories. The best way to spread information regarding a new tool is via word of mouth—we use those tools our peers swear by. Thus, in the spirit of advancing scientific progress, we submit a challenge to the research community: play with these tools, and evaluate their utility. If they're good, tell others. And if they aren't, tell the designers. This information age is an exciting time in that knowledge is at our fingertips. But if we fail to innovate upon our means of accessing information, the Internet's promise of providing us what we want will be lost as knowledge is drowned in a sea of facts. These new tools are founded upon the belief that we're better off working together, but they work only if you think so too.

